# Supplementary material for: “I Needed to Know, No Matter What I Do, I Won’t Make It Worse”—Expectations and Experiences of Last Aid Course Participants in Germany—A Qualitative Pilot Study
Source: Healthcare (Basel). 2023 Feb 16;11(4):592. doi: 10.3390/healthcare11040592 (PMC9956657; doi:10.3390/healthcare11040592)
Supplement: Supplementary file 1 [file healthcare-11-00592-s001.zip › healthcare-2146150-supplementary.pdf]

Informal care in outpatient end-of-life care using the example of Last Aid Courses - Pilot interview topic/questions guide

<u>I: Participation in the Last Aid Course</u>

General introduction

- Please tell me about your participation in a Last Aid Course.

Last Aid Course

- When did you participate in the Last Aid Course?
    - How did you find out about the courses?
    - How did you enroll in the courses? (Procedure of the registration)
    - With whom did you participate in the course? (relatives, partner, friend, etc.)
- For what reasons did you participate in the Last Aid Course?
    - What were your expectations for the course?
    - To what extent were these expectations met?
    - What would you have wished for?
- Overall, how did you like participating in the Last Aid Course?

<u>II: Competence for action</u>

- What content did you take away from the course?
- What helped you (especially)?
    - What did you actually implement in practice?
    - What else would you have wished for?
- Were there or are there any other effects you noticed from participating in the course?

<u>III: Outlook</u>

- What recommendations do you have for the course?
    - What would you change about the course?
    - What would you wish for other course participants?

<u>IV: Closing</u>

- I have now asked all the questions that are important to me. Do you perhaps have anything else that we haven't discussed yet, but that you would like to address?

The original pilot interview topic guide is in German, as the interviews were conducted in German. For comprehensibility of the methodology, therefore it is translated into english.
